# Perinatal Stress and Methamphetamine Exposure Decreases Anxiety-Like Behavior in Adult Male Rats

**DOI:** 10.3389/fnbeh.2021.648780

**Published:** 2021-04-29

**Authors:** Anna Holubová-Kroupová, Romana Šlamberová

**Affiliations:** Department of Physiology, Third Faculty of Medicine, Charles University, Prague, Czechia

**Keywords:** methamphetamine, prenatal stress, postnatal stress, maternal separation, anxiety, open field, elevated plus maze

## Abstract

Methamphetamine (MA) is an illicit synthetic psychostimulant drug, and its abuse is growing worldwide. MA has been reported as the primary drug of choice, by drug-abusing women, during pregnancy. Since MA easily crosses the placental barrier, the fetus is exposed to MA in a similar fashion to the mother. This study aimed to evaluate the effect of long-term perinatal stressors and drug exposure on anxiety-like behavior in adult male rats using the open field test (OF) and elevated plus maze (EPM). Dams were divided into three groups according to drug treatment during pregnancy: controls (C), saline—SA [subcutaneous (s.c.), 1 ml/kg], and MA (s.c., 5 mg/kg). Litters were divided into four groups according to postnatal stressors: non-stressed controls (N), maternal separation (S), maternal cold water stress (W), and maternal separation plus maternal cold water stress (SW). Forty-five minutes before testing (in both OF and EPM), one-half of adult male rats received an (s.c.) injection of MA and the other half received an SA injection. Prenatal MA/stress exposure did not affect anxiety-like behavior in adult male rats in both tests. In the OF, an acute MA dose in adulthood increased the time spent in the central disk area, decreased time spent in the corners, and decreased time spent immobile and grooming. Also, postnatal stress increased time spent in the central disk area, decreased time spent in corners, and increased mobility compared to controls. All groups of rats exposed to postnatal stressors spent significantly less time in the closed arms of the EPM compared to controls. Overall, our results indicate that early postnatal stress and a single acute MA administration in adulthood decreases the parameters of anxiety-like behavior in adult male rats regardless of prenatal MA exposure. Moreover, postnatal stress *via* maternal separation impacts the effect of acute MA administration in adulthood. Long-term postnatal stress may thus result in improved adaptation to subsequent stressful experiences later in life.

## Introduction

Methamphetamine (MA) is an illicit synthetic psychostimulant drug, which is abused worldwide at an increasing rate. MA use remains a significant public health concern in the Czechia and many other countries (European Monitoring Centre for Drugs and Drug Addiction, 2019; United Nations Office on Drugs and Crime (UNODC), 2012; Courtney and Ray, 2014). Moreover, MA is reported as an illicit drug of choice during pregnancy (Marwick, 2000; O’Connor et al., 2020). Since MA easily crosses the placental barrier, the fetus is exposed to this drug without protection from maternal metabolic processing (Dattel, 1990). MA exposure during pregnancy is then associated with increased rates of psychosocial risks and premature births (Marwick, 2000; O’Connor et al., 2020). Prenatal MA exposure is associated with altered function of the hypothalamic–pituitary–adrenal (HPA) axis, which may lead to some behavioral problems such as altered arousal in infants, increased emotional reactivity and anxiety, and depressive behavior in 3–5-year-old children (Zuloaga et al., 2015). Experimental studies have described various sequelae related to prenatal MA exposure. Our previous studies have demonstrated that prenatal drug exposure can impair sensorimotor development (Šlamberová et al., 2006; Holubová et al., 2017) as well as the development of the central nervous system of rat pups and induce various psychological impairments (Šlamberová, 2012; Macúchová et al., 2013). Although it was shown that prenatal MA exposure could induce changes affecting behavior during the preweaning period, these changes did not seem to persist into adulthood (Hrubá et al., 2010, 2012). On the other hand, a study by Bubeníková-Valešová et al. (2009) demonstrated that adult offspring prenatally exposed to MA or exposed to an acute MA injection in adulthood had higher baseline levels of dopamine in the nucleus accumbens. Moreover, there is at least one more factor that should not be overlooked, which is prenatal stress directly due to daily injection. Preclinical studies dealing with maternal injections during pregnancy have found that the injection itself, regardless of the injected substance (drug or saline), can induce long-term impairment of the stress response in adult offspring (Šlamberová et al., 2018).

Drug-addicted women live in some of the most socio-economically disadvantaged areas (O’Connor et al., 2020), which means their children are often confronted with socio-economic-related postnatal stressors. It was found that drug-addicted mothers who used MA during pregnancy reported more parenting stress and depression-positive diagnoses compared to controls (Liles et al., 2012; Smith et al., 2015). Stressful incidents during the early postnatal period are believed to be closely related to the development of psychological alterations and disorders such as depression, anxiety, attention deficit hyperactivity disorder (ADHD), and/or Alzheimer’s disease (Heim and Nemeroff, 2001; Aisa et al., 2008; Abrines et al., 2012; Briones et al., 2012). Chronic exposure to stress may impair locomotor and physiological responses to novel environments and induce anxiety-like behavior (Jankord et al., 2011; Taylor et al., 2016; Holubová et al., 2018a, b). Studies by Chung et al. (2005) and Maccari et al. (1995) found that maternal stress induced longer periods of corticosterone (CORT) secretion in rodents after a single acute stress exposure compared to controls. Thus, it has been suggested that repeated postnatal stress may activate the stress system, and prolonged CORT secretion is a result of a failure to properly control the stress response. This overexposure to stress hormones may then exacerbate a variety of chronic stress-induced diseases (McEwen, 2000; Chung et al., 2005). On the other hand, increased plasma levels of adrenocorticotropic hormone (ACTH) and decreased plasma levels of CORT were observed after acute stress exposure in rats previously exposed to chronic postnatal stressors compared to unstressed controls (Holubová et al., 2016).

Contradictory results were also shown in animal studies showing the effects of acute MA exposure on anxiety-like behavior in adulthood. Acute MA exposure may increase anxiety-like behavior in adult rats in the elevated plus maze (EPM) (Pometlová et al., 2012), while other studies showed decreased anxiety after acute MA exposure in the open field (OF) and the EPM (Herbert and Hughes, 2009; Schutová et al., 2009). Moreover, a clinical study by Nasirzadeh et al. (2013) found that drug-addicted young adults (humans) have signs of psychiatric disorders with more cases of depression, anxiety, and stress compared to the general population (Nasirzadeh et al., 2013).

As far as we know, there are no available studies examining the mechanisms and effects of the combination of both perinatal stress and MA abuse. A study by Gutierrez et al. (2017) found that repeated variable stress aggravates the effects of MA on monoamines through neuroinflammation and glutamatergic excitotoxicity, but it should be noted that the effects of stress events depend on the intensity, type, and length of the stressors. It was shown that a diverse set of stressors of low-to-moderate intensity could induce either positive or negative effects. Animals can even become more resilient to the stressor, and stress exposure may even attenuate MA neurotoxicity (Gutierrez et al., 2017).

**FIGURE 1:**
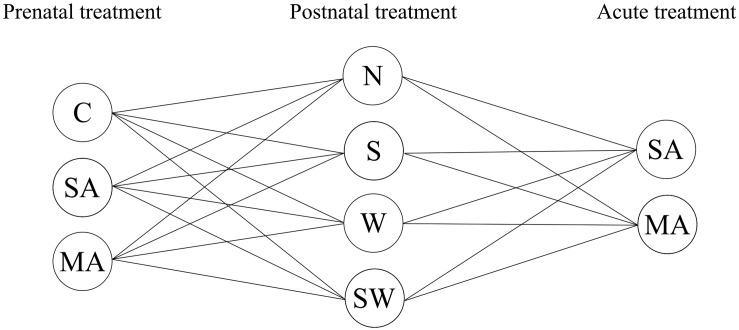
Overview of groups used in the present study. Prenatal treatment: C, control; SA, saline; MA, methamphetamine. Postnatal treatment: N, non-stressed controls; S, maternal separation; W, maternal cold water swimming stress; SW, maternal separation plus maternal cold water swimming stress. Acute SA indicates a single direct saline injection prior to testing, and acute MA indicates a single direct methamphetamine injection prior to testing.

The present study used a multifactorial experimental design resulting in 24 treatment groups (Figure 1). We used three groups of prenatal treatment (control, stress, and MA) and four groups of postnatal treatment (control, social stressor, physical stressor, and a combination of both). Moreover, on the final day of the experiment, all groups were divided in two individual groups with single acute MA or saline (SA) administration. In the present study, daily injection of SA [1 ml/kg, subcutaneous (s.c.)] during the pregnancy of rat dams was used to mimic prenatal stress, one of the potential influencing factors. Maternal separation (S) and maternal cold water stress (W) were used as models of early life stress. To the best of our knowledge, the current study is the first to investigate the effect of multiple long-term perinatal factors, stressors, and MA exposure on anxiety-like behavior in adult male rats. Additionally, we looked at possible drug sensitization in adulthood. This was accomplished by observations in the OF arena and the EPM. Overall, these findings may provide insight into behavioral changes induced by multiple perinatal factors, i.e., stress and/or MA in male adult rats.

## Materials and Methods

The procedures for animal experimentation utilized in this study were reviewed and approved by the Institutional Animal Care and Use Committee and are in agreement with the Czech Government Requirements under the Policy of Humans Care of Laboratory Animals (no. 246/1992) and with subsequent regulations of the Ministry of Agriculture of the Czechia (no. 311/1997).

### Prenatal Care

Adult female albino Wistar rats were delivered by Velaz (Prague, Czechia) from Charles River Laboratories International, Inc., Females (weight 250–300 g) were housed in groups of four per cage and left undisturbed for 1 week in a temperature-controlled (22–24°C) colony room with free food and water on a 12-h (light)/12-h (dark) cycle (with lights on at 6:00 am). After a week of acclimatization, dams were housed overnight with mature males (Šlamberová et al., 2006). Females were also randomly assigned to those who would receive, daily during the entire gestation period, (1) a subcutaneous (s.c.) injection of MA (5 mg/kg); (2) SA (1 ml/kg), and (3) controls (C) without any injection. On day 20 of gestation, dams were placed into maternity cages alone. The day of delivery was taken as postnatal day (PD) 0.

### Postnatal Care

**FIGURE 2:**
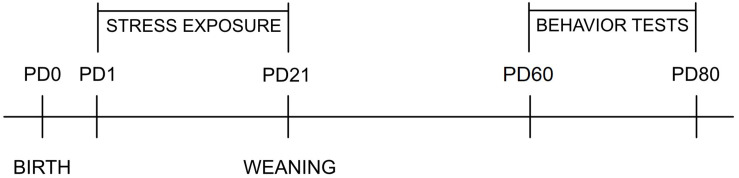
The timetable of experiments. PD, postnatal day.

The total number of litters was 68. Litter size was adjusted to 12 pups, and pups were cross-fostered starting on PD 1, so that one mother usually raised four control, four SA, and four MA pups. For recognition, prenatally, MA-exposed pups were injected (intradermally) with black India ink in the left foot, SA-exposed pups in the right foot, and controls were not tattooed. Equal numbers of males and females were raised by each mother whenever possible. Litters were divided into four groups: non-stressed controls (N), maternal separation (S), maternal cold water stress (W), and maternal separation plus maternal cold water stress (SW). Stress exposure was conducted once daily starting on PD 1 and ending on PD 21 when they were weaned. Six animals from each litter were used—always one control, one SA, and one MA for the OF and the EPM tests, respectively. The rest of the animals were used in other studies. The timetable of experiments is presented in Figure 2.

#### Social Stress

Maternal separation as a social stressor was conducted from PD 1–21 for 3 h per day between 8:00 and 11:00 am (Huot et al., 2004; Plotsky et al., 2005; Lajud et al., 2012; Holubová et al., 2016). All pups from the appropriate groups, i.e., S and SW, were gently removed from their maternity cages and placed in separate cages in another room for 3 h. The cages with pups were always placed on heating pads to prevent hypothermia.

#### Physical Stress

Maternal cold water stress served as the physical stressor (Drago et al., 1999; Šlamberová et al., 2002; Holubová et al., 2017). Stress exposure of this stressor was conducted once daily from PD 1 to PD 21 of pups. A plastic container (25 cm × 35 cm × 40 cm, *L* × *W* × *H*) was filled with 5°C water to a depth of 25 cm. Rat mothers from the appropriate groups, i.e., W and SW, were forced to swim in cold water for 5 min. Rats were then towel-dried, placed carefully under a heating lamp until they were mostly dry, and then returned to their cages. The water in the containers was cleaned after each animal.

#### Acute Dose in Adulthood

Before OF and EPM observations, rats were divided into two groups based on acute drug administration. Forty-five minutes prior to testing, one-half of the adult male rats received (s.c.) a single direct injection of MA (1 mg/kg) and the other half a single direct injection of SA (1 ml/kg).

### Behavioral Tests

The behavior of the adult male offspring (PD 60–80) was tested in OF (total number of 200 animals, *n* = 8–10 per group) and EPM (total number of 206 animals, *n* = 8–10 per group) (Schutová et al., 2009, 2010). To avoid an impact of other acute injection or multiple contact with drug in adulthood or previous test, no animal was used in both tests. Rats were not habituated to either the OF or EPM apparatus so that the exposure was to that of an unfamiliar environment during the tests. On the day of the experiment, rats were moved to the testing room, where they remained in their home cage for a 30-min acclimation period. The rat’s behavior was recorded on video for a 10-min period in the OF arena and for a 5-min period in the EPM. After that, rats were removed, and the floor and walls of the testing apparatus were cleaned using Ajatin solution and dried thoroughly before the next animal was tested. This was to avoid the influence of trace smells on behavior.

The OF arena (45 cm × 45 cm × 30 cm) was enclosed by three opaque walls and a transparent front wall. The floor of the OF arena was crisscrossed with lines, and in the center was drawn a central disk (20 cm in diameter). Video recordings were evaluated manually by using ODLog software. The measured parameters were as follows: (1) locomotion, i.e., the number of lines crossed; (2) time spent in the central disc; (3) time spent in the corners; (4) sniffing time; (5) rearing time; (6) grooming time; and (7) time spent immobile.

The EPM consisted of four interconnected arms elevated 50 cm above the floor. Two of the opposite arms were enclosed by opaque plastic walls while the other two arms were opened. Since all arms were connected to a central open area, rats could freely move among the arms. The measured parameters in the EPM were as follows: (1) time spent in the closed arms; (2) time spent in the open arms; (3) frequency of the stretched attend posture (SAP), which means stretching the head and shoulders into a novel or threatening area, followed by a quick traction of the head and shoulders to their original position; (4) sniffing time; and (5) rearing time.

### Statistics

D’Agostino–Pearson omnibus normality test and Shapiro–Wilk normality test were performed to test data for normality. No significant results suggested that the data is normally distributed. Thus, a three-way ANOVA (prenatal × postnatal × acute treatments) and two-way ANOVA (prenatal × postnatal; prenatal × acute; postnatal × acute treatments) were used to analyze all parameters of the adult male rats. The Bonferroni test was used for *post hoc* test comparisons. Differences were considered significant if *p* < 0.05. Data were expressed on graphs as mean ± SEM.

## Results

### Open Field

#### Anxiety

**FIGURE 3:**
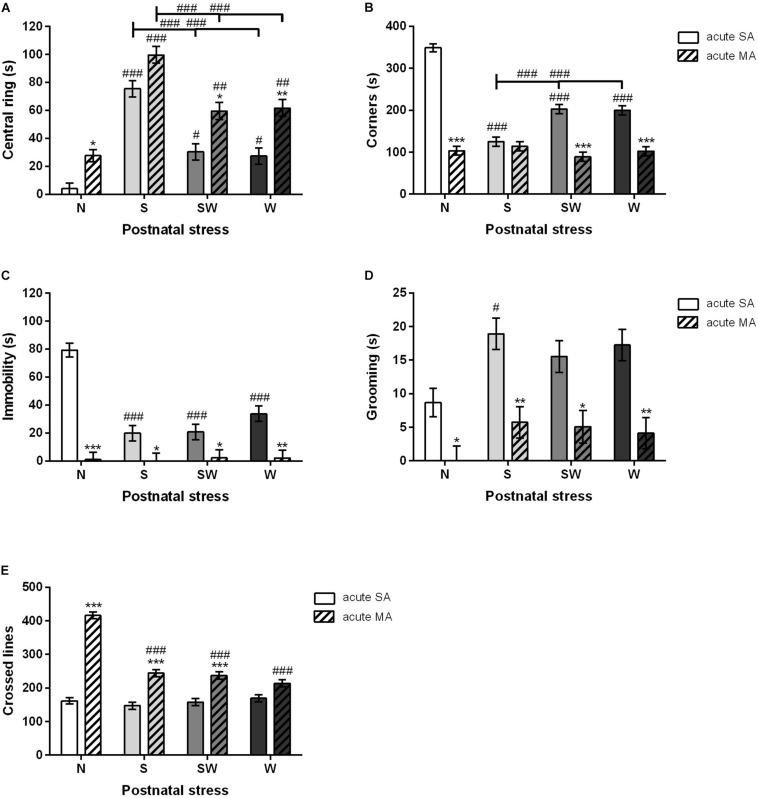
The effect of postnatal stress and acute MA administration on anxiety- like behavior in the open field test. **(A)** Time spent in the central ring, **(B)** time spent in the corners, **(C)** time spent immobile, **(D)** grooming time, and **(E)** the number of lines crossed. Values are means ± SEM. N, non-stressed controls; S, maternal separation; W, maternal cold water swimming stress; SW, maternal separation plus maternal cold water swimming stress. Acute SA indicates a single direct saline injection prior to testing, and acute MA indicates a single direct methamphetamine injection prior to testing. *p < 0.05, **p < 0.01, ***p < 0.001 significant difference in one group of *x*-axis. ^#^*p* < 0.05, ^##^*p* < 0.01, ^###^*p* < 0.001 significant differences between the group in the legend and its N control.

A multifactorial ANOVA was conducted that examined the effect of prenatal, postnatal, and acute treatments. Early postnatal stress and acute MA administration prior to testing significantly impacted all observed parameters. Both these factors, postnatal stress [*F*_(3, 192)_ = 57.23; *p* < 0.001] and acute treatment [*F*_(1, 192)_ = 50.91; *p* < 0.001], were statistically significant in the first parameter “time spent in the central disc.” The Bonferroni *post hoc* test revealed that S (*p* < 0.001), SW (*p* < 0.05), and W (*p* < 0.05) groups increased the time spent in the central disk of the OF arena compared to non-stressed rats N. Moreover, acute MA dose in adulthood also increased time spent in the central disk compared to SA counterparts, N (*p* < 0.05), SW (*p* < 0.05), and W (*p* < 0.01). No differences were found in the postnatal S group (Figure 3A). On the other hand, rats exposed to early postnatal stress [*F*_(3, 192)_ = 39.55; *p* < 0.001], i.e., S (*p* < 0.001), SW (*p* < 0.001), and W (*p* < 0.001), spent less time in the corners of the OF arena than non-stressed controls (N). The acute MA dose in adulthood [*F*_(1, 192)_ = 232.70; *p* < 0.001] decreased the time spent in the corners compared to SA rats, with regard to postnatal stress, N (*p* < 0.001), SW (*p* < 0.001), and W (*p* < 0.001). No differences were found in the postnatal S group (Figure 3B).

Postnatal stress [*F*_(3, 192)_ = 13.39; *p* < 0.001] decreased the time spent immobile in all stressed groups, i.e., S (*p* < 0.001), SW (*p* < 0.001), and W (*p* < 0.001), compared to non-stressed rats N. Acute MA dose in adulthood [*F*_(1, 192)_ = 93.54; *p* < 0.001] further decreased immobile time, which was abolished across all groups, i.e., N (*p* < 0.001), S (*p* < 0.05), SW (*p* < 0.05), and W (*p* < 0.01) (Figure 3C). Acute MA administration [*F*_(1, 192)_ = 48.41; *p* < 0.001] also decreased the time spent grooming (licking and face washing) in all postnatal groups, i.e., N (*p* < 0.05), SW (*p* < 0.05), S (*p* < 0.01), and W (*p* < 0.01), compared to an acute SA injection prior to testing. Postnatal stress [*F*_(3, 192)_ = 4.59; *p* < 0.05] revealed a significant difference in group S that spent more time grooming (*p* < 0.05) than non-stressed controls (N) (Figure 3D).

#### Locomotion

Significant differences were shown by both factors, postnatal stress [*F*_(3, 192)_ = 40.21; *p* < 0.001] and acute treatment in adulthood [*F*_(1, 192)_ = 255.0; *p* < 0.001], in locomotion, where rats from the N (*p* < 0.001), S (*p* < 0.001), and SW (*p* < 0.001) postnatal groups exposed to the acute MA injection in adulthood crossed more lines than the rats with the acute SA injection. Interestingly, postnatal stress significantly decreased the impact of acute MA in all postnatally stressed groups (*p* < 0.001) compared to non-stressed controls (N) (Figure 3E).

#### Rearing and Sniffing

**FIGURE 4:**
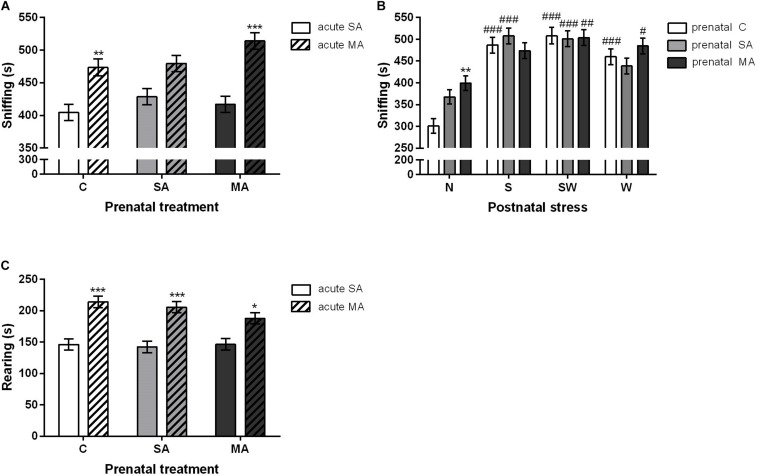
The effect of perinatal treatment and acute MA administration on sniffing and rearing behavior in the open field test. **(A)** Sniffing time, **(B)** sniffing time, and **(C)** rearing time. Values are means ± SEM. C, control; SA, saline; MA, methamphetamine; N, non-stressed controls; S, maternal separation; W, maternal cold water swimming stress; SW, maternal separation plus maternal cold water swimming stress. Acute SA indicates a single direct saline injection prior to testing, and acute MA indicates a single direct methamphetamine injection prior to testing. *p < 0.05, **p < 0.01, ***p < 0.001 significant differences in one group on the *x*-axis. ^#^*p* < 0.05, ^##^*p* < 0.01, ^###^*p* < 0.001 significant differences between the group in the legend and its N control.

Similarly, a statistical analysis of rearing and sniffing revealed the significant effect of acute MA treatment and postnatal stress exposure. Acute MA injection [*F*_(1, 194)_ = 49.40; *p* < 0.001] increased the time spent sniffing in the prenatal MA (*p* < 0.001) group and prenatal controls (C) (*p* < 0.01) compared to acute SA rats (Figure 4A). Statistical analysis also showed a significant interaction between prenatal and postnatal treatment [*F*_(6, 188)_ = 2.78; *p* < 0.05]. Prenatally, MA-exposed rats in the group of non-stressed controls spent more time sniffing (*p* < 0.01) compared to prenatal controls (Figure 4B). An acute MA dose prior to testing induced significant differences [*F*_(1, 192)_ = 49.41; *p* < 0.001] in the amount of sniffing time with regard to postnatal treatments, in group S (*p* < 0.001) and W (*p* < 0.001). There were no differences in the non-stressed group of rats as well as in the most stressed postnatal group, i.e., SW, after an acute MA injection compared to their SA counterparts. Postnatal stress [*F*_(3, 192)_ = 42.25; *p* < 0.001] increased sniffing time in all postnatally exposed groups (*p* < 0.001) compared to non-stressed controls. With regard to rearing, there were statistically significant differences between groups exposed to acute injection of MA/SA in adulthood [*F*_(1, 194)_ = 60.44; *p* < 0.001]. A Bonferroni *post hoc* test revealed an increased rearing in all prenatal groups, i.e., C (*p* < 0.001), SA (*p* < 0.001), and MA (*p* < 0.05), despite the acute MA treatment compared to the acute SA groups. Of interest was the lower significance in the prenatal MA-exposed rats compared to the prenatal C and SA groups. However, this effect was found only in the postnatally non-stressed group of rats (Figure 4C).

### Elevated Plus Maze

#### Anxiety

**FIGURE 5:**
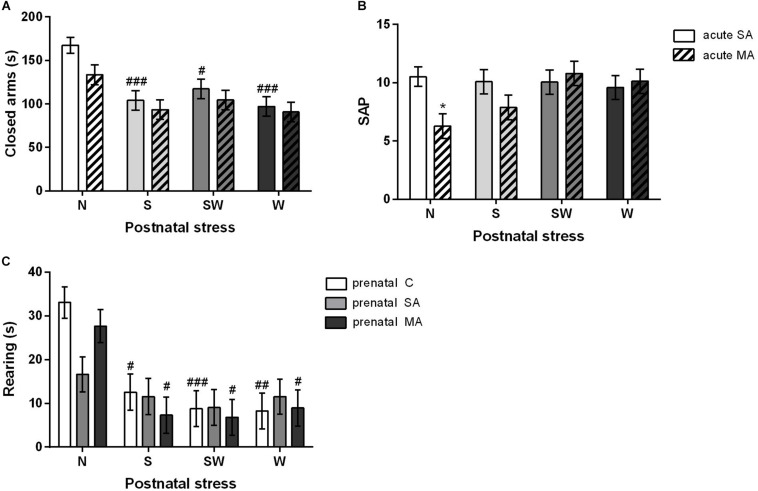
The effect of postnatal treatment and acute MA administration on anxiety-like behavior in elevated plus maze test. **(A)** Time spent in the closed arms, **(B)** frequency of the stretched attend posture, and **(C)** rearing time. Values are means ± SEM. C, control; SA, saline; MA, methamphetamine; N, non-stressed controls; S, maternal separation; W, maternal cold water swimming stress; SW, maternal separation plus maternal cold water swimming stress. Acute SA indicates a single direct saline injection prior to testing, and acute MA indicates a single direct methamphetamine injection prior to testing. *p < 0.05, **p < 0.01, ***p < 0.001 significant differences in one group on the *x*-axis. ^#^*p* < 0.05, ^##^*p* < 0.01, ^###^*p* < 0.001 significant differences between the group in the legend and its N control.

There were statistically significant differences in postnatal treatment [*F*_(3, 197)_ = 10.81; *p* < 0.001]. *Post hoc* test using the Bonferroni correction revealed that all postnatally affected groups, S (*p* < 0.001), SW (*p* < 0.05), and W (*p* < 0.001), spent less time in the closed arms compared to non-stressed rats N (Figure 5A). There were no differences in the time spent in the open arms. There was a significant interaction between the effects of postnatal and acute treatments [*F*_(3, 198)_ = 2.73; *p* < 0.05] in SAP parameter. Postnatal stress mitigated the effect of acute MA dose on SAP parameter seen in the non-stressed group N (*p* < 0.05), i.e., there were no differences between the acute MA and SA groups in any of the postnatally stressed groups (Figure 5B).

#### Rearing and Sniffing

With regard to rearing in the EPM, the two-way ANOVA that examined the effect of postnatal and acute treatment showed significant differences in both factors, postnatal [*F*_(3, 198)_ = 12.10; *p* < 0.001] and acute [*F*_(3, 198)_ = 9.03; *p* < 0.01] treatments. Postnatal stress (*p* < 0.05) decreased the time spent rearing after acute MA administration compared to non-stressed controls (N). The two-way ANOVA examining the effect of prenatal and postnatal treatment revealed significant differences in postnatal treatment only [*F*_(3, 194)_ = 12.46; *p* < 0.001]. Interestingly, prenatally SA-exposed rats showed nearly the same levels across all postnatal groups without any significant differences. By contrast, postnatal stress in the prenatal C and prenatal MA groups led to decreased rearing time; in S group (both *p* < 0.05), in SW group (*p* < 0.001 and *p* < 0.05), and in W group (*p* < 0.01 and *p* < 0.05), respectively (Figure 5C). There were no differences in the time spent sniffing.

## Discussion

### Anxiety

The present data showed that prenatal treatments, i.e., MA and stress exposure, have no effect on anxiety-like behavior in adult male rats measured using the OF and EPM tests. These findings are in agreement with previous studies where daily MA injections (5 mg/kg) during rat pregnancy did not reveal any significant differences related to behavioral activity in adulthood (Schutová et al., 2009; Hrubá et al., 2012). A study by Hrubá et al. (2012) evaluated changes resulting from chronic prenatal and postnatal exposure of MA in adult male rats, and they found that only postnatal exposure to MA *via* breastfeeding from rat mothers affected anxiety-like behavior in adult male rats (Hrubá et al., 2012). On the other hand, a study by Bubeníková-Valešová et al. (2009) found increased baseline levels of dopamine in the nucleus accumbens (288% increase) in MA prenatally exposed adult male rats. The higher levels of dopamine were then associated with less time spent immobile and a bigger response to the challenge dose of MA given during adulthood compared to SA rats (Bubeníková-Valešová et al., 2009).

An acute MA dose in adulthood significantly decreased the parameters of anxiety-like behavior in both the OF and EPM tests. A challenge dose of MA 45 min before testing increased the time spent in the central disk of the OF arena, decreased the time spent in OF corners, decreased the time spent immobile, and decreased the grooming time. These findings agree with previous studies (Herbert and Hughes, 2009; Schutová et al., 2009). The study by Schutová et al. (2009) found that the same acute dose of MA (1 mg/kg) administrated prior to testing decreased the time spent in the OF corners, decreased the time spent immobile, and increased the time spent in the OF central disk (Schutová et al., 2009). Some of these effects were not seen in the postnatal S group, which experienced maternal separation, where baseline levels of given activities did not differ from those seen after acute SA administration. Although the time spent in both types of arms (open/closed) did not differ, acute MA decreased the frequency of SAP in the EPM, which is in agreement with a study by Schutová et al. (2009). This effect was seen only in the postnatally non-stressed rats, i.e., group N. There is, however, a study that found acute MA exposure increased anxiety-like behavior in the open field compared with SA controls (Struntz and Siegel, 2018). Nevertheless, this study used adult mice as subjects; the injection of MA was immediately prior to behavior testing; and a higher dose of MA (4 mg/kg) was used, which has the potential to induce stereotypical behaviors (Struntz and Siegel, 2018), all of which is in contrast to the present study, which used a lower dose of MA (1 mg/kg) that was administered 45 min prior to testing the adult rats. Thus, it seems that the effect of acute MA on anxiety-like behavior depends on the amount of MA administered and the interval between administration and testing.

All postnatal stressors used in the present study decreased the parameters of anxiety-like behavior in the OF arena, i.e., postnatal stress increased the time spent in the central disc, decreased the time spent in the corners, and decreased the immobile time compared to non-stressed controls (N). Moreover, all groups of rats exposed to a postnatal stressor spent significantly less time in the closed arms of the EPM compared to non-stressed controls (N). Interestingly, rat offspring postnatally exposed to the social stressor only (separation) (S) showed no differences in the total time spent in the central disk and corners after acute MA administration compared to the SA group. It seems that the social stressor, i.e., maternal separation 3 h per day, changes the sensitivity to acute methamphetamine in adulthood. However, available data describing the effects of postnatal stress on anxiety-like behavior are varied. Some studies have shown an increase in anxiety-like behavior after neonatal maternal separation (de Melo et al., 2018; Jin et al., 2018). A study by de Melo et al. (2018) found that adult male, but not female, rats spent less time in the open arms and more time in the closed arms of the EPM. The study also reported decreased dendritic spine density in the prefrontal cortex (dorsal agranular insular cortex—AID) and increased densities in the hippocampus CA1 area in adult male rats (de Melo et al., 2018). However, our data support the results from a study by Yang et al. (2019), suggesting that rats exposed to stress early in life, *via* daily maternal separation from PD 2 to PD 14 for 3 or 6 h, had decreased anxiety-like behaviors (Yang et al., 2019). Our data agree with a study by Borges-Aguiar et al. (2018), where 3 h of daily maternal separation during the entire lactation period failed to affect anxiety behaviors in adult rats in the EPM or OF. The reduction or lack of anxiogenic effects in postnatally stressed rats in adulthood is also in accordance with other studies that used different tests to investigate anxiety-like behavior (e.g., fear conditioning, social interaction, and restraint stress) (McIntosh et al., 1999; Caldji et al., 2000; Hulshof et al., 2011; Savignac et al., 2011). These studies suggest that stressful events during early life periods can result in adaptive behaviors (Sachser et al., 2011; Mittal et al., 2015). Despite this, severe stressful events may result in detrimental effects, while perinatal low-to-moderate stressors may induce protective effects in adulthood (Yang et al., 2019). For example, a brief daily separation of pups from their mother during the early postnatal period can reduce the effects of chronic stress on the HPA axis reactivity (Oines et al., 2012). This is in agreement with our previous data where increased plasma levels of ACTH and decreased plasma levels of CORT were observed in postnatally stressed (S and SW) groups of rats after acute stress exposure compared to unstressed controls (N) (Holubová et al., 2016). Long-term postnatal stress may thus lead to a lower sensitivity of ACTH receptors in the adrenal cortex, which may result in improved adaptation to subsequent stressful experiences. Chronic stress in early life periods thus may prepare the nervous, immune, and endocrine systems to cope with stressful experiences later in life (Grissom and Bhatnagar, 2009; Daskalakis et al., 2011; Holubová et al., 2016; Yang et al., 2019).

The types and levels of the early postnatal stressors we studied were not stimulating MA neurotoxicity in male offspring in adulthood. Some studies have demonstrated that chronic variable stressors enhance MA-induced neurotoxicity (Tata and Yamamoto, 2008; Northrop and Yamamoto, 2012) as well as responsivity to this psychostimulant (Matuszewich et al., 2014; Anderson et al., 2019). However, this effect was only shown in groups of rats receiving an acute injection of a high dose of MA (7.5 mg/kg) compared to non-stressed controls (Matuszewich et al., 2014; Anderson et al., 2019). The group of rats receiving an acute MA injection of 1 mg/kg, which was the same dose used in our present study, did not show any differences (Anderson et al., 2019). Moreover, the effect seems to strongly depend on the types of stressors used. Some previous studies demonstrated that a chronic moderate stressor provided a mild protective effect against MA-induced striatal astrogliosis, enhanced specific types of cognitive performance during the Morris water maze (MWM) test, such as target acquisition (Gutierrez et al., 2017), and improved spatial memory in the MWM as well as enhanced memory during the novel object recognition tests (Parihar et al., 2011), suggesting that the animals adapted to consequent stress exposure.

### Other Behavioral Activities

Acute MA significantly increased psychomotor activity in groups N, S, and SW, i.e., it increased the number of lines crossed (locomotion) in the OF, which is in accordance with other studies (Schutová et al., 2010; Struntz and Siegel, 2018). There was no significant difference between group W exposed to the acute MA injection in adulthood and group W exposed to the acute SA injection. Nevertheless, there were no significant changes among all postnatal groups. Further experiments focusing on these stressors are planned. Moreover, a challenge dose of MA in adulthood significantly increased the parameters of exploratory activities relative to prenatal treatment. Regarding rearing, groups that were prenatally exposed to MA, after receiving a challenge dose of the same drug in adulthood, reared less than those prenatally exposed to SA and controls (C). This supports a previous study (Schutová et al., 2010) where an acute dose of MA significantly increased the time spent by rearing of rats prenatally exposed to saline, while it did not affect the group with prenatal MA exposure. However, this effect, as it related to rearing, was only found in the non-stressed group of rats (N). Postnatal stress raised baseline levels of rearing time in all groups, but acute MA dose did not significantly elevate the measured levels in the OF arena. Interestingly, prenatally SA-exposed rats showed nearly the same low levels across all groups, and postnatal stress only affected prenatal groups C and MA, which showed decreased rearing time in the EPM. Regarding sniffing, prenatally stressed (SA-exposed) rats showed no difference after acute dose of MA in adulthood. This is in contrast to prenatal controls and prenatally MA-exposed rats that showed significant differences after a challenge dose of MA. Prenatally MA-exposed rats in the group of non-stressed controls (N) spent also more time sniffing compared to prenatal controls (C). Thus, our study supports a previous hypothesis that long-term prenatal MA exposure may be associated with higher sensitivity to the same drug administered directly during adulthood, even though it was only a single acute exposure (Bubeníková-Valešová et al., 2009; Schutová et al., 2009).

### Conclusion

It should be emphasized that stress can be adaptive or maladaptive depending on the type, severity, and length of exposure. Overall, our results indicate that early postnatal stress and a single acute MA administration in adulthood decrease the parameters of anxiety-like behavior in adult male rats regardless of prenatal exposure. However, early postnatal stress impacts the effect of acute MA administration. Prenatal stress and prenatal drug exposure can change the exploratory activities of rats to novel environments in adulthood. Since MA abuse among humans continues to rise worldwide, more detailed studies with distinct setups are needed to better understand the mechanism and the potential human impact of MA abuse, especially during pregnancy.

## Data Availability Statement

The original contributions presented in the study are included in the article/supplementary material, further inquiries can be directed to the corresponding author/s.

## Ethics Statement

The animal study was reviewed and approved by the Institutional Animal Care and Use Committee and are in agreement with the Czech Government Requirements under the Policy of Humans Care of Laboratory Animals (No. 246/1992) and with subsequent regulations of the Ministry of Agriculture of the Czechia (No. 311/1997).

## Author Contributions

AH-K is responsible for the experimental parts, video analysis, and statistical analysis, as well as for the present manuscript. RŠ is a supervisor of AH-K and the head of the department and the laboratory where this study has been conducted. RŠ has been involved in all parts of the present study. Both authors contributed to the article and approved the submitted version.

## Conflict of Interest

The authors declare that the research was conducted in the absence of any commercial or financial relationships that could be construed as a potential conflict of interest.
